# Melatonin Relations With Respiratory Quotient Weaken on Acute Exposure to High Altitude

**DOI:** 10.3389/fphys.2018.00798

**Published:** 2018-06-29

**Authors:** Marcelo Tapia, Cristian Wulff-Zottele, Nicole De Gregorio, Morin Lang, Héctor Varela, María Josefa Serón-Ferré, Ennio A. Vivaldi, Oscar F. Araneda, Juan Silva-Urra, Hanns-Christian Gunga, Claus Behn

**Affiliations:** ^1^Owl Capacitaciones y Asesorías SpA, Antofagasta, Chile; ^2^Facultad de Ciencias de la Salud, Universidad de Antofagasta, Antofagasta, Chile; ^3^Facultad de Medicina, Instituto de Ciencias Biomédicas, Universidad de Chile, Santiago, Chile; ^4^Facultad de Ciencias Básicas, Universidad de Antofagasta, Antofagasta, Chile; ^5^Facultad de Medicina, Universidad de los Andes, Santiago, Chile; ^6^Center for Space Medicine and Extreme Environments, Institute of Physiology, Charité – Universitätsmedizin Berlin, Berlin, Germany; ^7^Facultad de Medicina, Universidad San Sebastián, Santiago, Chile

**Keywords:** melatonin, circadian rhythm, high altitude, respiratory quotient, body timekeeping

## Abstract

High altitude (HA) exposure may affect human health and performance by involving the body timing system. Daily variations of melatonin may disrupt by HA exposure, thereby possibly affecting its relations with a metabolic parameter like the respiratory quotient (RQ). Sea level (SL) volunteers (7 women and 7 men, 21.0 ± 2.04 y) were examined for daily changes in salivary melatonin concentration (SMC). Sampling was successively done at SL (Antofagasta, Chile) and, on acute HA exposure, at nearby Caspana (3,270 m asl). Saliva was collected in special vials (Salimetrics Oral Swab, United Kingdom) at sunny noon (SMC_D_) and in the absence of blue light at midnight (SMC_N_). The samples were obtained after rinsing the mouth with tap water and were analyzed for SMC by immunoassay (ELISA kit; IBL International, Germany). RQ measurements (*n* = 12) were realized with a portable breath to breath metabolic system (Oxicon^TM^ Mobile, Germany), between 8:00 PM and 10:00 PM, once at either location. At SL, SMC_D_, and SMC_N_ values (mean ± SD) were, respectively, 2.14 ± 1.30 and 11.6 ± 13.9 pg/ml (*p* < 0.05). Corresponding values at HA were 8.83 ± 12.6 and 13.7 ± 16.7 pg/ml (n.s.). RQ was 0.78 ± 0.07 and 0.89 ± 0.08, respectively, at SL and HA (*p* < 0.05). Differences between SMC_N_ and SMC_D_ (SMC_N_–SMC_D_) strongly correlate with the corresponding RQ values at SL (*r* = -0.74) and less tight at HA (*r* = -0.37). Similarly, mean daily SMC values (SMC_
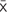
_) tightly correlate with RQ at SL (*r* = -0.79) and weaker at HA (*r* = -0.31). SMC_N_–SMC_D_, as well as, SMC_
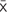
_ values at SL, on the other hand, respectively, correlate with the corresponding values at HA (*r* = 0.71 and *r* = 0.85). Acute exposure to HA appears to loosen relations of SMC with RQ. A personal profile in daily SMC variation, on the other hand, tends to be conserved at HA.

## Introduction

Contemporary working conditions, tend to challenge the human body internal timing system. Jet-lag (Coste et al., 2004), and extreme environments (Arendt, 2012; Najjar et al., 2014), affect circadian rhythms. Circadian misalignment sets the basis for metabolic disorders and cell cycle alterations that ultimately implicate risks at work and disease (Archer et al., 2014; Smolensky et al., 2016; Swanson et al., 2016). Circadian deregulation on high altitude (HA) exposure (Mortola, 2007, 2017) added to desynchronization by shift-work (Andlauer et al., 1979; Reinberg and Ashkenazi, 2008; Mirick et al., 2013) may well represent a factor involved in the lethal outcome of remote HA mining (Zaldívar Larraín, 2013).

Living beings synchronize with periodic environmental challenges. Various *Zeitgebers*, among them the daily light/dark cycle synchronize endogenous time keepers, the biological clocks (Reddy and O’Neill, 2010; Thut et al., 2012; Tsang et al., 2013). Rhythms result from a changing balance between activators and repressors in a negative feedback loop or between synthesis and degradation rates of oscillator components (Pulivarthy et al., 2007; see also Li et al., 2017). Intersecting with cellular biochemistry, multiple oscillators finally yield physiological, and behavioral rhythms (Top and Young, 2017). Countless oscillators, with widely differing oscillation periods, constitute the body timing system (Beale et al., 2016; Tran et al., 2016). Interacting among themselves (Schroeder and Lakatos, 2009; Zelano et al., 2016), the oscillators represent temporal reference frames for each other (Thut et al., 2012; Thurley et al., 2017). A complex information handling framework thus results (Rapp, 1987; Lloyd and Rossi, 1993).

Melatonin synchronizes cellular clocks with its own epiphyseal secretion, the latter being driven, via suprachiasmatic nuclei (Coomans et al., 2013), by the daily light/dark cycle determined by Earth rotation (Arendt, 1996; Chakir et al., 2015; Hardeland, 2015). Rhythm synchronization integrates body functions, both by local (Lin et al., 2017), as well as, by systemic means (Pfeffer et al., 2017). Melatonin (*N*-acetyl-5-methoxytryptamine), an ubiquitous, pleiotropic, and multitasking indoleamine (for recent reviews see Luchetti et al., 2010; Reiter et al., 2010; Hardeland et al., 2012) derives from tryptophan successively being transformed into serotonin and *N*-acetylserotonin. An *N*-acetyltransferase, involved in melatonin synthesis, is inhibited by light. Melatonin, thus, acts as a chemical transmitter of darkness (Tan et al., 2010; Hardeland et al., 2011). The non-image-forming vision system entraining body function rhythmicity via melatonin also implicates a subpopulation of retinal ganglion cells (ipRGCs ≈ 1% of the retinal ganglion cell population; Panda et al., 2003). The ipRGCs depolarize in response to photostimulation (Berson et al., 2002). Melanopsin, the photopigment of ipRGCs, absorbs light at aprox. 480 nm, the wavelength most effective in suppressing melatonin secretion (for a recent review see Lucas et al., 2014). Notably, melanopsin is also present in epithelial cells of the lens (Alkozi et al., 2017).

Melatonin involvement in overall circadian regulation relates to energy metabolism (Peschke et al., 2013; Cipolla-Neto et al., 2014) including termoregulation (Gubin et al., 2006; Kräuchi et al., 2006) and redox status (Maciel et al., 2010; Jiménez-Ortega et al., 2012; Tan et al., 2013; Cudney et al., 2014) acting, among others, as a natural antioxidant (Nehela and Killiny, 2018). Melatonin targets genes (Unfried et al., 2010; Hardeland et al., 2011; Torres-Farfán et al., 2011), the epigenome (Korkmaz et al., 2012; Haim and Zubidat, 2015), as well as, mitochondria (Acuña-Castroviejo et al., 2003; Maciel et al., 2010).

High altitude exposure may affect melatonin rhythm by lack of oxygen. Hypoxia, the lack of oxygen as related to aerobic energy requirements (Connett et al., 1990), delays the phase of melatonin rhythm (Coste et al., 2009). Untreated obstructive sleep apnoea syndrome, a clinical condition implicating intermittent hypoxia, leads to an early morning plateau of plasma melatonin concentration. This morning plateau of melatonin is reversed into a night time peak by increasing oxygen supply via CPAP device application in treated obstructive sleep apnoea patiernts (Hernández et al., 2007). Hypoxia applied for two hours in a hypobaric chamber (simulating 8,000 m a.s.l.) increases plasma melatonin concentration in rats (Kaur et al., 2002). This body timing system, thus, may be alterated by an environmental challenge such as a rapid ascent from sea level (SL) up to 3,000 m a.s.l., as usual in Chilean Andes. Respiratory quotient (RQ) elevation on HA exposure indicates an increase of glucose utilization under that condition. Insulin-regulated pathways depend on integrity of biological clocks (McGinnis et al., 2017). We, thus, examined effects of acute exposure at HA on the circadian rhythm of the chronotropic neurohormone melatonin and its relation with a metabolic parameter like RQ, the latter representing, a point of reference for energy metabolism at HA.

## Materials and Methods

### Subjects

Fourteen healthy volunteers (**Table 1**), all of them students enrolled in Physical Education Pedagogy at University of Antofagasta, volunteered for the present study in the context of a wider HA research project (FONDECYT 1100161). Having previously been approved by the Ethics Committee of the Faculty of Medicine, University of Chile, the latter project was also endorsed by Bioethical Committee of Faculty of Health Sciences, University of Antofagasta, considering the principles and practices stated in the Declaration of Helsinki for studies of human beings. A written informed consent was obtained from each subject finally participating in the study.

**Table 1:** Body dimensions of the volunteers.

Physical parameters of the volunteers (mean ± SD)				

	Age (years)	Weight (kg)	Height (cm)	Body mass index (kg/m^2^)
Women (*n* = 7)	21.7 ± 2.63	64.7 ± 12.8	163 ± 3.40	24.2 ± 4.48
Men (*n* = 7)	20.3 ± 0.95	71.6 ± 5.77	174 ± 6.07	23.8 ± 3.02
Total (*n* = 14)	21.0 ± 2.04	68.1 ± 10.2	169 ± 7.19	24.0 ± 3.68


*Parameters measured at SL.*

### Study Design

The volunteers were examined for salivary melatonin concentration (SMC) at SL, the site of their usual residence. Cardio-respiratory parameters could be obtained in only 12 of them (**Table 2**). Corresponding measurements at HA were done in the context of a pedagogic field trip, on the day after arriving by bus, at Caspana (3,270 m a.s.l.), a small village located in the Andes, 300 km east from Antofagasta.

**Table 2:** Cardio-respiratory parameters at SL (Antofagasta) and HA (Caspana, 3,270 m a.s.l.).

Cardio-respiratory parameters of the volunteers (*n* = 12)		

	SL	HA
HR (beats/min)	67.1 ± 9.55	90.3 ± 12.1*
VE (l/min)	9.54 ± 1.19	15.4 ± 4.56*
BR (breaths/min)	15.3 ± 4.27	25.9 ± 5.56*
VO_2_ (ml/min)	300.3 ± 42.6	376.2 ± 86.7*
VCO_2_ (ml/min)	233.8 ± 39.9	336.2 ± 96.0*
HbO_2_ sat (%)	99.7 ± 0.47	95.8 ± 1.07*
RQ	0.78 ± 0.07	0.89 ± 0.08*


*The asterisk denotes the difference between SL and HA values being significant (*p* < 0.05). HR, heart rate; VE, pulmonary ventilation; BR, breathing rate; VO_*2*_, oxygen flux; VCO_*2*_, carbon dioxide flux; HbO_*2*_ sat, hemoglobin oxygen saturation; RQ, respiratory quotient.*

### Measurements

#### Salivary Melatonin Concentration

At SL, as well as, at the HA site, the subjects were required to provide saliva samples for SMC determination, with sun light at midday (SMC_D_) and dim, ordinary bulb light, at midnight (SMC_N_). After rinsing the mouth with tap water, samples of saliva (1.5 ml aprox.) were collected into special vials (Salimetrics Oral Swab, United Kingdom), The saliva samples were handled using gloves, coded and stored in liquid nitrogen, to be later on analyzed for SMC with an ELISA kit (IBL International, Germany) in an independent commercial laboratory (Red Lab S.A., Santiago, Chile). SMC_N_–SMC_D_ and SMC_
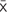
_ are, respectively, assumed to represent the amplitude of daily SMC change and the average of both day and night SMC value per subject.

#### Respiratory Quotient

Cardio-respiratory parameters were determined under resting conditions, after sitting for 5 min. The measurements were done between 8:00 and 10:00 PM, both at SL and HA, once at either location. Evening meals consisted of bread and cheese at SL, as well as at HA. Along a 3 min equilibration period, respiratory CO_2_ and O_2_ fluxes could be measured in 12 of the 14 subjects with a portable metabolic system, including a breath-to-breath spirometer (Oxicon^TM^ Mobile, Germany). RQ was calculated as the ratio between mean CO_2_ flux and mean O_2_ flux.

### Statistics

Mean values are expressed ± SD. ANOVA for repeated measurements was applied for comparisons between SMC_D_ and SMC_N_ at SL and HA. Pearson’s correlation coefficient and Student’s *t*-test were, respectively, applied for analysis of correlations and for comparison between SL and HA. Calculations were done with the aid of SPSS 22 IBM software package. Statistic significance was established at the *p* < 0.05 level.

## Results

**FIGURE 1:**
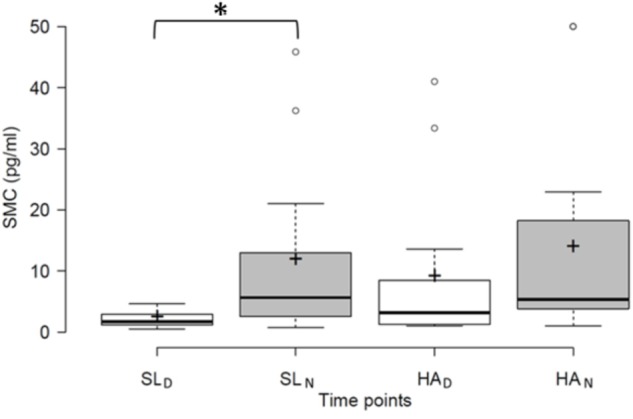
SMC at SL_D_, SL_N_, HA_D_, and HA_N_. Subindices D and N, respectively, indicate whether the samples were obtained at day or at night. The black line in the boxes denotes the median. Mean values are represented by a cross. White circles indicate outlier values. Asterisks denote that differences are significant (*p* < 0.05).

**FIGURE 2:**
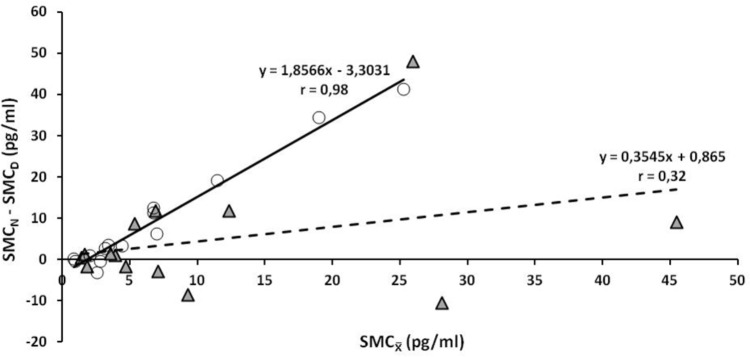
SMC_N_–SMC_D_ vs. SMC_
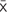
_ values at SL and HA. Simple linear regression of the relation between SMC_N_–SMC_D_ and SMC_
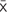
_ at SL (white circles) and HA (gray triangles), are depicted with continuous and dashed line, respectively.

**FIGURE 3:**
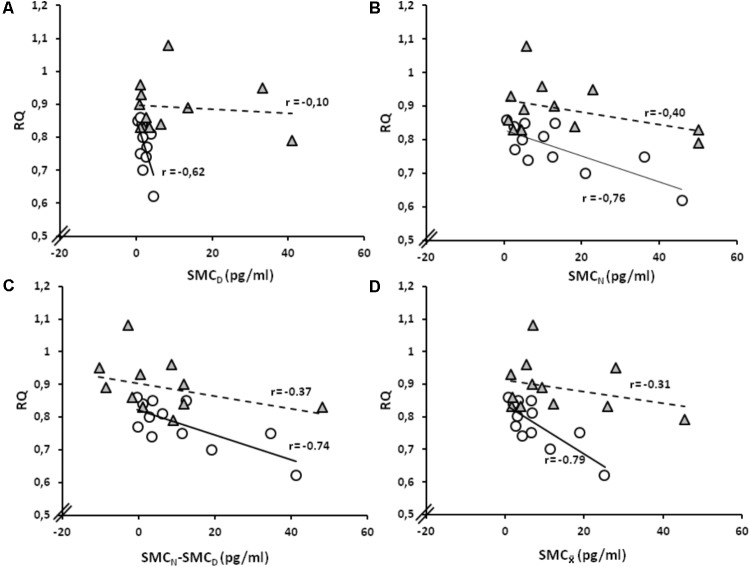
SMC values as related to RQ. **(A)** Simple linear regression of the relation between RQ and SMC_D_ at SL and HA. **(B)** Simple linear regression of the relation between RQ and SMC_N_ at SL and HA. **(C)** Simple linear regression of the relation between RQ and SMC_N_–SMC_D_ at SL and at HA. **(D)** Simple linear regression between RQ and SMC_
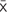
_ at SL and HA. White circles and gray triangles, respectively, represent SL and HA values (*n* = 12).

Age and body mass index were rather similar in women and men volunteering in the present study (**Table 1**). Cardio-respiratory parameters of the volunteers significantly changed on HA exposure as compared to SL (**Table 2**). **Figure 1** shows mean values of SMC at day and night, both at SL (SL_D_, SL_N_) and HA (HA_D_, HA_N_). Mean SMC values in either those conditions were similar in women and men (data not shown). Daily variations of SMC observed at SL vanish at HA. SMC_N_–SMC_D_ and SMC_
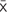
_, respectively, depict, for the present work, the amplitude of circadian melatonin rhythm and the average value around which the oscillation occurs. These parameters strongly correlate one with the other at SL. At HA, on the contrary, this correlation weakens (**Figure 2**). RQ–SMC_D_ relation appears to be strong at SL and weak at HA (**Figure 3A**). Similarly, the RQ–SMC_N_ relation appears to be tighter at SL than at HA (**Figure 3B**). Both, SMC_N_–SMC_D_ (**Figure 3C**), as well as, SMC_
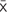
_ (**Figure 3D**), also correlate with RQ more strongly at SL than at HA

**FIGURE 4:**
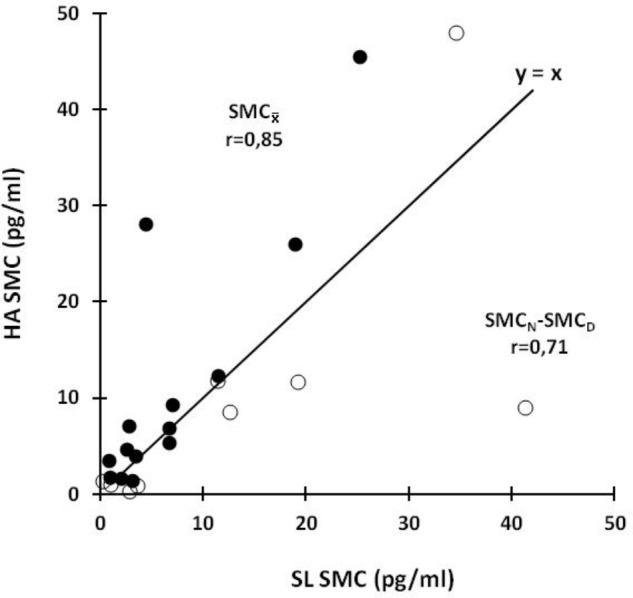
SMC_N_–SMC_D_ and SMC_
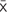
_ values at SL and at HA. Identity line is depicted as a continuous black line. Black circles show the relation between SL and HA SMC_
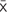
_ values. White circles show the relation between SL and HA SMC_N_–SMC_D_ values.

SMC_
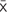
_ values at SL strongly correlate with those at HA (black circles, **Figure 4**). Similarly, SMC_N_–SMC_D_ at SL also tightly correlate with the corresponding values at HA (white circles, **Figure 4**). As also shown in **Figure 4**, the former and the latter relation, respectively, locate mainly above and below the middle line (*y* = *x*).

## Discussion

Mean SMC_N_ and SMC_D_ values differ at SL but not at HA (**Figure 1**). SMC_N_ and SMC_D_, as well as, SMC_N_–SMC_D_ and SMC_
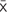
_, correlate with RQ strongly at SL and much less so at HA (**Figure 3**). Melatonin circadian rhythm, thus, may lose at HA its synchronizing grip on aspects related with energy metabolism. Individual SMC_
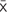
_ and SMC_N_–SMC_D_ values at SL, on the other hand, strongly correlate with the corresponding ones at HA (**Figure 4**). Although being distorted at HA (**Figures 1**–**3**), an individual profile of circadian melatonin rhythmicity, thus, seems to persist under the latter condition (**Figure 4**). Such an individual profile of circadian melatonin rhythmicity may in the future be explored for its potential to predict the capacity for adequately dealing with challenges of the body timing system.

Salivary melatonin has been validated as an adequate marker for phase typing of circadian regulation (Voultsios et al., 1997). Although representing only one third of plasma melatonin concentration (Benloucif et al., 2008), SMC adequately relates to the latter (Voultsios et al., 1997). Hyposalivation and low melatonin levels may limit the reliability of SMC, as measured by radioimmunoassay in the elderly (Gooneratne et al., 2003). Liquid chromatography combined with mass spectrometry, on the other hand, revealed SMC values to exceed free plasma melatonin concentration on average by 36% (van Faassen et al., 2017). Like in oral mucosa (Chaiyarit et al., 2017), melatonin may be also locally produced in salivary glands (van Faassen et al., 2017). Whether related or not with plasma melatonin, SMC shows in the present work a clear rhythmicity, that may even represent changes occurring at tissue level. An ELISA kit used in the present work yielded SMC values very similar to those reported by others (Lushington et al., 2002; Verheggen et al., 2012).

Mean SMC_N_ and SMC_D_ values differ at SL but not at HA (**Figure 1**). SMC_N_–SMC_D_ considered, in the present study, as the amplitude of daily melatonin variation, correlates with SMC_
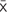
_ (the average value around which the oscillation occurs) more strongly at SL than at HA (**Figure 2**). Distortions of SMC rhythm as shown to occur at HA (**Figures 1**, **2**) may implicate a deregulation of melatonin-dependent periodic processes. High amplitudes in circadian melatonin rhythmicity may prevent and/or delay the development of diabetes (Hardeland, 2017). The amplitude of daily melatonin oscillation, on the other hand, diminishes in the elderly (Gubin et al., 2006; Kim et al., 2014).

Disruption of body timekeeping, implicates deregulation of body functions (Cipolla-Neto et al., 2014; O’Neill and Feeney, 2014). Three weeks of circadian disruption induce a pre-diabetic condition in otherwise healthy subjects (Buxton et al., 2012). Energy metabolism unbound from circadian pacemakers associates to obesity, diabetes, cardiovascular disease, and cancer (Miller et al., 2010; Blask et al., 2014; Zubidat and Haim, 2017). SMC_N_–SMC_D_, as well as, absolute values of SMC_N_ and SMC_D_ loosening their relation with RQ at HA (**Figure 3**) could mean a decoupling of energy metabolism from circadian control, a possibility that certainly has further to be elucidated. It may be noticed, however, that even acute adequation of energy metabolism to HA exposure is yet far from reaching a consensus (Chicco et al., 2018). It may be provisionally assumed, however, that mistiming of melatonin circadian rhythmicity may represent a metabolic risk factor, particularly under conditions combining shift work with hypoxia as being usual in Chilean Andes.

Deregulation of circadian melatonin rhythmicity may result from changes in oxygen supply. Hypoxia also implicates an increase in sympathetic activity. Sympathetic afferent nerves of the pineal gland activate an *N*-acetyltransferase, the rate-limiting enzyme for melatonin synthesis. Beta-blockers, older age and a higher body mass, on the other hand, have been found to lower nocturnal urinary 6-sulfatoxymelatonin levels (Davis et al., 2001). Melatonin secretion may, moreover, additionally be altered at HA by hypocapnia prevailing in newcomers at HA. Neurons of suprachiasmatic nucleus are, in fact, particularly sensitive to pH (Chen et al., 2009).

Individual values of SMC_N_–SMC_D_ and SMC_
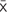
_ observed at SL, respectively, correlate with the corresponding value at HA (**Figure 4**). Individual patterns in melatonin circadian rhythmicity as observed at SL, thus, appear largely to be conserved at HA. Individual circadian melatonin rhythmicity seems, indeed, to remain relatively stable (Fernández et al., 2017). With exception of sedation and/or artificial ventilation (Olofsson et al., 2004), neither activity, posture, sleep, nor menstrual phase appear to affect individual circadian rhythm of melatonin (Cain et al., 2010). From one subject to another one, nocturnal melatonin concentration can, on the other hand, differ considerably (Zeitzer et al., 1999). Some people seem to be able to rapidly modify their melatonin secretion pattern, as well as, to readily adapt to rotating shift schedules (Quera-Salva et al., 1997). Similarly, physiological adjustments to acute HA exposure vary, indeed, substantially from one subject to another. Individual characteristics of circadian melatonin rhythmicity, yet to be defined, may well relate with the capacity to adequately deal with challenges of the body timing system affecting energy metabolism in health and disease.

To summarize, a rapid ascent to an altitude of about 3,000 m a.s.l., as usual under working conditions in the Andes, tends to override the night-day difference of SMC and to weaken the relations between SMC with RQ, thus, potentially deregulating melatonin-dependent timing of body functions, affecting energy metabolism. Individual SL circadian profile of SMC tends, on the other hand, to be maintained at HA. The SL profile of melatonin circadian rhythm may be further on explored for its potential to predict individual tolerance to challenges of the body timing system at HA.

## Author Contributions

CB, CW-Z, JS-U, ML, and MT were mostly implicated in the experimental design, logistics, and development of the experimental work at SL and HA. MT, CW-Z, NDG, ML, HV, MS-F, EV, OA, JS-U, H-CG, and CB substantially contributed to the conception of the work, data analysis and manuscript revision, approved the final version, and agree to be accountable for the whole work. The M.Sc. thesis of MT at the Faculty of Health Sciences, University of Antofagasta, is mainly based on this work.

## Conflict of Interest Statement

The authors declare that the research was conducted in the absence of any commercial or financial relationships that could be construed as a potential conflict of interest.
